# Serological evidence of Lassa virus in commensal rodents from Senegal

**DOI:** 10.1186/s12879-026-13450-z

**Published:** 2026-05-02

**Authors:** Seynabou Seye, Mouhamed Kane, Safiétou Sankhe, Ndongo Dia, Gamou Fall, Oumar Faye, Mawlouth Diallo, Alioune Gaye, Moussa Moise Diagne

**Affiliations:** 1 Virology Department, Institut Pasteur de Dakar, Dakar, 220 Senegal; 2 Zoology Medical Department, Institut Pasteur de Dakar, Dakar, 220 Senegal

**Keywords:** Lassa virus, Rodent reservoirs, Invasive species, One health surveillance, Zoonotic emergence, Senegal

## Abstract

**Background:**

Lassa fever, a neglected zoonotic hemorrhagic disease caused by Lassa virus (LASV) and endemic in West Africa, remains a significant public health concern associated with rodent exposure. Senegal lies at the western fringe of the LASV area, but only one 1988 serosurvey reported low antibody prevalence. Given recent ecological shifts, including the expansion of invasive *Rattus rattus* and *Mus musculus*, we reassessed LASV exposure in rodents from Senegal.

**Methods:**

We retrospectively analyzed 618 archived rodent sera collected in 2012–2013 from domestic and peri-domestic environments in central and eastern Senegal. Samples were screened for LASV-specific IgG by ELISA, and IgM was assessed descriptively in IgG-positive or equivocal specimens, with cautious interpretation because rodent validation for IgM remains limited. Spatial mapping and ecological analysis identified seropositivity clusters and potential environmental correlates.

**Results:**

Eleven rodents were IgG-seroreactive (1.8%; 95% CI: 0.9–3.2%), and no IgM reactivity was detected, although IgM results were interpreted cautiously because rodent validation remains limited. All seroreactive animals belonged to the commensal species *Rattus rattus* (9/180; 5%) and *Mus musculus* (2/174; 1.1%) and were clustered in five villages along major transport corridors. At the locality level, overall positivity ranged from 3.3% to 28.6%, with the highest values observed in Didé Gassama (26.3%) and Kounkane (28.6%), indicating a focal rather than diffuse pattern of LASV exposure.

**Conclusions:**

This study provides the first update in over three decades on LASV exposure in Senegalese rodents. IgG seroreactivity confined to invasive commensal species suggests localized exposure patterns that warrant further investigation, while the weak, non-significant association between rodent diversity and seropositivity does not support a clear diversity effect. Spatial clustering of seropositive rodents along major transport routes points to low-level but persistent circulation in settings favoring human–rodent contact. These findings provide a retrospective baseline of LASV seroreactivity in Senegalese rodents and highlight the need to integrate rodent surveillance into One Health frameworks to strengthen early warning and regional preparedness.

**Clinical trial number:**

Not applicable.

**Supplementary Information:**

The online version contains supplementary material available at 10.1186/s12879-026-13450-z.

## Introduction

Lassa fever is an endemic viral hemorrhagic disease in parts of West Africa and represents an important emerging infectious disease threat. Lassa fever virus (LASV) was first identified in 1969 and is classically associated with the Natal multimammate mouse *Mastomys natalensis*. However, molecular and phylogenetic studies have shown that LASV can also be detected in other small mammals, notably *Mastomys erythroleucus*,* Hylomyscus pamfi*, and more recently in *Mus baoulei* and *Lophuromys sikapusi*, in specific West African settings, suggesting that LASV ecology may involve locally heterogeneous multi-host dynamics rather than a strictly single-host system [1–5]. Human infections occur primarily via rodent-to-human spillover, emphasizing the importance of rodent ecology in disease control. LASV is endemic to West Africa and causes thousands of human cases each year [6].

Senegal lies at the western edge of LASV’s known distribution. Although no cases have been documented within its borders, Eastern Senegal shares frontiers with endemic areas of Mali and Guinea and is subject to regular cross-border movement of people and goods, creating opportunities for invasive species introductions and pathogen spread.

Notably, previous surveys in neighboring countries have documented LASV in rodents. In Guinea, for example, seroprevalence up to 27–50% of tested *M. natalensis* had LASV antibodies, depending of the studies [7–9], as well as occasional spillover to other rodent species. In southern Mali, nearly 20% of *M. natalensis* were LASV-positive (by antibody or antigen) and all were found in village homes [10]. Similarly, recent work in Nigeria found broadly distributed LASV seroprevalence (up to ~ 50% in *M. natalensis*) and even occasional positives in other species such as *M. erythroleucus* and *R. rattus* [11].

In contrast, LASV has never been definitively isolated in Senegal although earlier serosurveys suggested possible exposure. A report published in 1988 described surveys of 1,440 rodents across the country, including villages where suspected cases had previously been reported [12]. The study found an overall LASV antibody prevalence of 1.2%, with positives detected mainly in *Mastomys* spp. (2.1%), *Arvicanthis niloticus* (0.7%), and *Mus musculus* (1.4%). Since that time, ecological changes such as the inland expansion of the invasive black rat (*Rattus rattus*), may have altered the composition of potential reservoirs. To update knowledge on LASV exposure risk along the Senegalese frontier and to examine community-level correlates, we analyzed archived rodent sera collected during 2012–2013 in Senegal as a retrospective baseline dataset.

## Methods

### Rodent sampling

Archived rodent sera were retrieved from the Institut Pasteur de Dakar (IPD) biobank and screened for LASV antibodies. The samples originated from field activities conducted within the broader CHANCIRA project, an interdisciplinary One Health program investigating the relationships between environmental change, circulation of people and goods, black-rat expansion, and zoonotic risk in Senegal. Rodent sampling for the present study was conducted between 2012 and 2013 in 26 localities in eastern, southeastern, and central Senegal, including sites along the Tambacounda–Kédougou and Tambacounda–Kidira axes, as well as additional villages in Kaffrine and Kaolack to extend spatial coverage. These areas were selected to provide broad spatial coverage of localities relevant to the CHANCIRA field framework, including major transport axes and human settlements. At regional and local scales, the field framework relied on a spatialized database integrating the geographic coordinates of villages and hamlets together with information collected at the level of concessions and households, including characterization of domestic and peri-domestic environments. Rodents were trapped in human settlements using Sherman and locally adapted traps in domestic (inside houses and other buildings) and peri-domestic (surroundings of concessions) settings. Trapping sessions generally lasted 1–5 consecutive days. In the broader commensal rodent trapping framework from which these samples derive, indoor trapping typically involved multiple rooms per locality, usually with two traps per room and daily trap checking and rebaiting. Captured animals were identified to species level as previously described [13]. Trapped small mammals were handled humanely in accordance with Senegalese legislation and relevant guidelines, euthanized by cervical dislocation after capture, and then processed for morphometric recording and dissection. Sera were archived at − 80 °C in the IPD biobank until testing.

### Serology testing and rodent diversity

Samples were assayed for LASV-specific IgG using Panadea Diagnostics ELISA kits (nucleoprotein antigen; Panadea Diagnostics GmbH, Hamburg, Germany) validated for rodent surveillance in West Africa [14]. Rodent validation has been more clearly established for IgG detection in *M. natalensis* and *M. erythroleucus* than for the other species included in this study. IgM assays were performed only descriptively on specimens that were IgG-positive or initially equivocal; however, because IgM has not been formally validated in rodents, these results were interpreted cautiously and were not used to infer acute infection. According to the manufacturer’s instructions, immunoreactivity values (IV) were classified as positive (IV ≥ 1.10), negative (IV ≤ 0.90), or equivocal (0.90 < IV < 1.10). Equivocal samples were retested, and IgM assays were performed only on specimens that were IgG-positive or initially equivocal. Each assay plate included manufacturer-provided controls to ensure assay validity, and experimental runs that failed to meet quality-control criteria were systematically repeated.

Species counts and prevalence estimates were generated with 95% confidence intervals (CIs) calculated using the exact Clopper–Pearson method.

Shannon diversity indices (H′) were derived from species counts for each site, and diversity was compared between LASV-seropositive and LASV-seronegative localities using the Mann–Whitney U test. Associations between species identity and LASV seropositivity were examined with Fisher’s exact test, with odds ratios (ORs) and exact p-values reported.

## Results

**Fig. 1:**
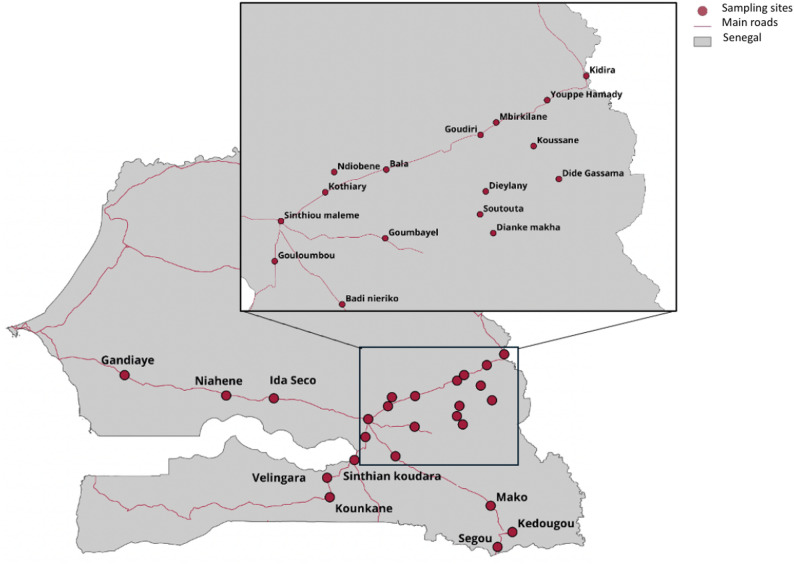
Geographic distribution of rodent sampling sites in Senegal (2012–2013). The map showing the 26 localities (red dots) where rodents were trapped for LASV serological screening. The inset highlights the eastern corridor (Tambacounda–Kédougou axis), where most captures occurred

A total of 618 archived sera were found. The geographic distribution of sampling sites is shown in Fig. 1. A supplementary locator map situating Senegal within West Africa and relative to neighboring countries with documented Lassa fever activity is provided in Supplementary Figure S1. This map was prepared using regional context adapted from Moore et al. [15], who modeled Lassa fever incidence across West Africa.

Collection localities were spread across central and southeastern Senegal, with sites in Kaffrine (Niahène and Ida Seco) and Kaolack (Gandiaye) as well as in Tambacounda, Kédougou (Kedougou, Segou, Mako) and Kolda(Velingara, Sintian koudara and Kounkane). They cluster specifically around transport corridors. The map highlights the wide coverage of the survey, spanning both central regions and border areas close to Mali and Guinea, where cross-border movement and human–rodent interactions are frequent.

Most sera originated from eastern Senegalese localities situated along the trans-Sahelian road near the Malian border, with Youppe Hamady (48/618; 7.8%), Kothiary (47/618; 7.6%), Dianké Makha (46/618; 7.4%), Kidira (45/618; 7.3%), and Bala (44/618; 7.1%) together accounting for nearly 37% of all samples. These eastern sites were sampled across both rainy and dry seasons, capturing contrasting ecological contexts and periods of rodent activity. Additional contributions came from central Senegal, particularly the Kaffrine villages of Niahène (34/618), and Ida Seco (28/618), as well as Gandiaye (29/618) in Kaolack, during the same seasonal campaigns. In contrast, fewer samples were obtained from more peripheral southeastern sites such as Kounkane, Kedougou, and Mako. Thus, our dataset reflects stronger representation of roadside and cross-border hubs compared with more remote areas, while spanning both wet- and dry-season conditions (see Figure S2). The corresponding serological results are presented in the Lassa infection subsection below.

A total of ten taxa were identified among the 618 individuals captured. Commensal species were most abundant, with *Rattus rattus* (*n* = 180; 29.1%) and *Mus musculus* (*n* = 174; 28.2%) together comprising over half of all captures (Figure S3), followed by *Crocidura* spp. (*n* = 137; 22.2%). Other species such as *Arvicanthis niloticus* (*n* = 53; 8.6%), *Mastomys erythroleucus* (*n* = 35; 5.7%), *Praomys daltoni* (*n* = 24; 3.7%) and *Mastomys natalensis* (*n* = 11; 1.8%) occurred at lower frequencies, while additional taxa (*Steatomys* sp., *Mus* (*Nannomys) spp*, *Gerbilliscus gambianus*) were only sporadically detected.

**Fig. 2:**
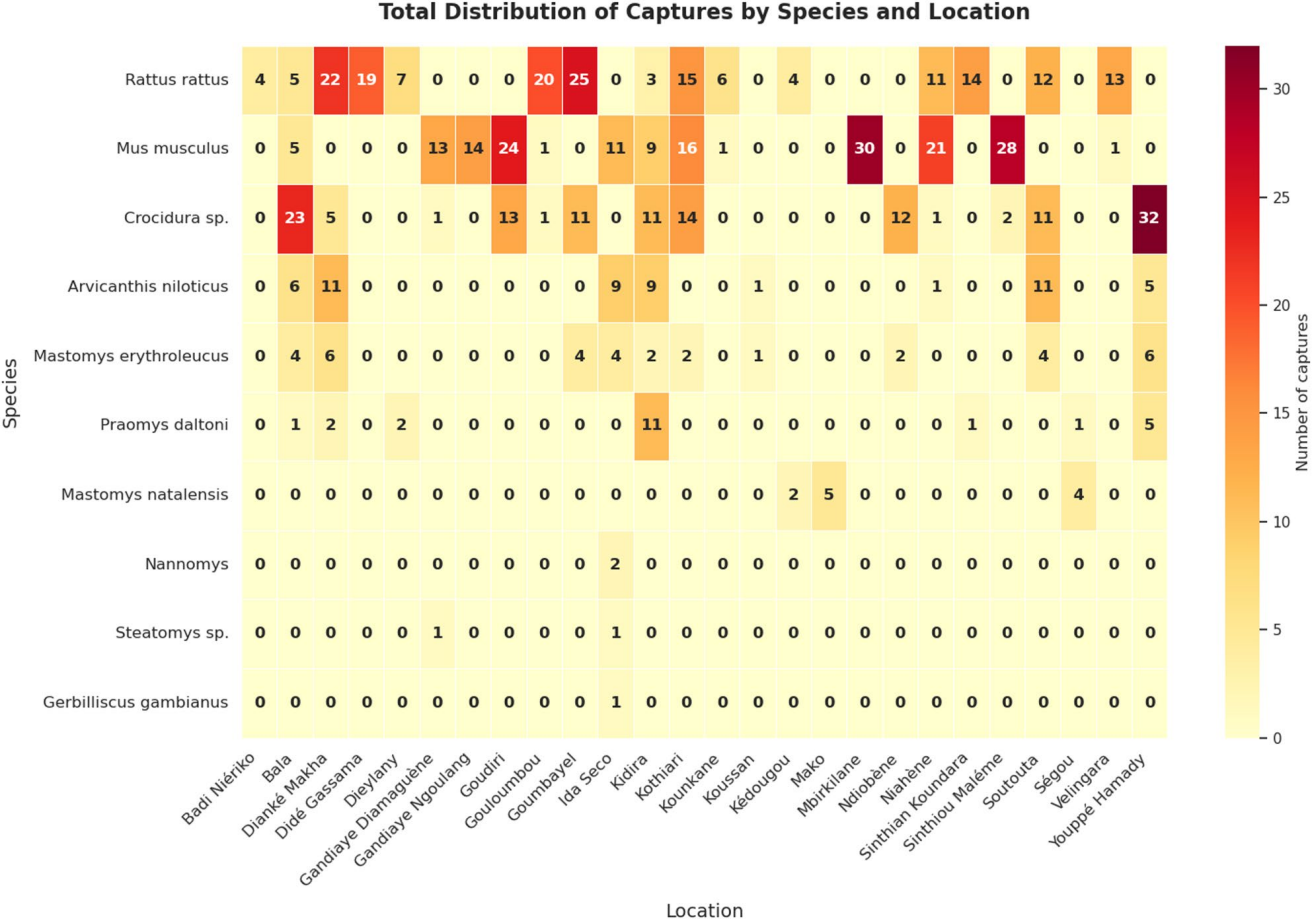
Distribution of captured rodent species across collection sites in Senegal (2012–2013). The heatmap showing the number of individuals captured per species and locality. *Rattus rattus* and *Mus musculus* were the most abundant and widely distributed species, particularly in roadside and cross-border villages such as Goumbayel, Dianké Makha, Gouloumbou, Didé Gassama, Mbirkilane, Sinthiou Malème, Goudiri, and Niahène. In contrast, *Mastomys natalensis*—the recognized LASV reservoir—was rare and found only in southeastern sites (Kédougou, Mako, and Ségou)

*R. rattus* was widely distributed, with higher numbers recorded in Goumbayel, Dianké Makha, Gouloumbou and Dide Gassama, while *M. musculus* showed a similarly broad distribution, particularly in Mbirkilane, Sinthiou Malème, Goudiri, and Niahène (Fig. 2). By contrast, *Mastomys natalensis* was rare and detected only in the southeastern sites: Kédougou, Mako and Segou. Overall, *R. rattus* and *M. musculus* were dominant and widespread, especially in roadside villages, whereas recognized LASV reservoir species were found only occasionally and in low numbers.

Sex ratios varied across species captured (Figure S4). *Rattus rattus* (57.5% females) and *Mus musculus* (59.8% females) showed significant female bias, while *Crocidura* sp., *Arvicanthis niloticus*, *M. erythroleucus*, and *M. natalensis* displayed more balanced distributions. *Praomys daltoni* also showed a female predominance (68.2%), whereas rare taxa (*Gerbilliscus gambianus*, *M*. (*Nannomys) spp*, *Steatomys* sp.) were represented by very few individuals, limiting interpretation.

### Lassa infection

**Fig. 3:**
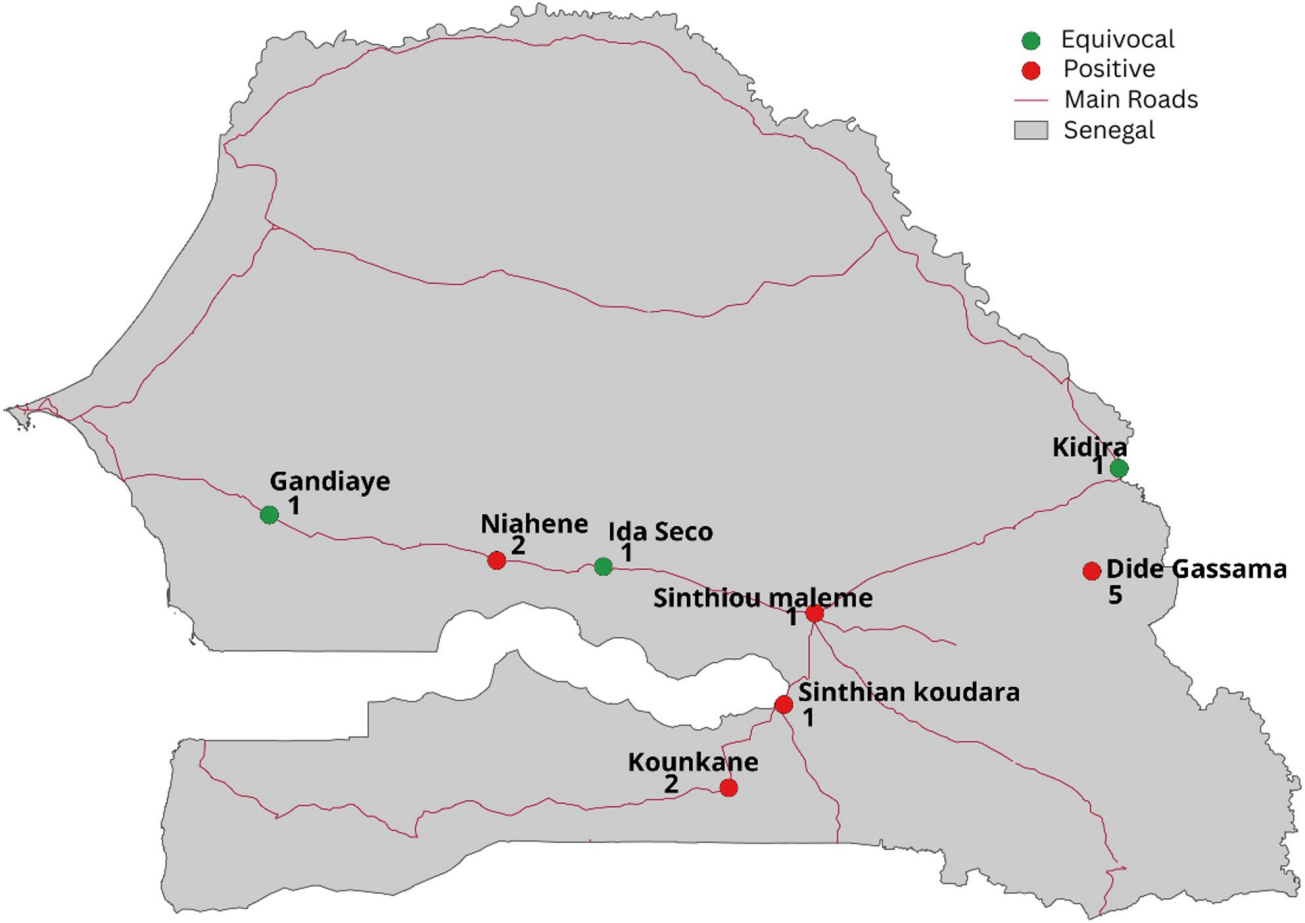
Spatial distribution of LASV IgG–positive (red) and equivocal (green) rodent samples in Senegal (2012–2013)

Of the 618 sera tested, 11 (1.8%; 95% CI: 0.9–3.2%) were positive for LASV-specific IgG (Table 1). All seropositive samples originated from commensal species, namely *R. rattus* (9/180; 5%) and *M. musculus* (2/174; 1.1%). No IgM reactivity was detected among the tested specimens. However, because IgM has not been formally validated in rodents, this finding was interpreted descriptively and not as evidence excluding recent infection. Although females were more frequently captured in *Rattus rattus* and *Mus musculus*, the small number of seroreactive animals did not allow robust assessment of sex-specific differences in LASV seroreactivity. Positivity was highly focal at the locality level along major transport and border corridors (Fig. 3), ranging from 3.3% in Sinthiou Maléme to 28.6% in Kounkane, with intermediate values of 5.9% in Niahène, 6.7% in Sinthian Koundara, and 26.3% in Didé Gassama. At the species-by-locality level, positivity reached 26.3% in *R. rattus* at Didé Gassama and 33.3% in *R. rattus* at Kounkane, showing that pooled species-level prevalence masks substantial local heterogeneity.

The corresponding IV for positive specimens ranged from approximately 1.12 to 3.82 (mean = 1.93 ± 0.95) (Table 1, Figure S5). Three additional sera (0.5%) yielded equivocal results (mean IV = 0.944 ± 0.045), indicating sporadic intermediate antibody reactivity. These were detected in *A. niloticus* from Ida Seco and in *M. musculus* from Gandiaye and Kidira.

**Table 1:** Summary statistics of LASV IgG ELISA results in rodents from Senegal (2012–2013). IV: Index Value

Serological status	*n*	Mean IV	Standard deviation	Median	Min. IV	Max. IV
Equivocal	3	0.944	0.045	0.942	0.901	0.991
Negative	604	0.285	0.114	0.254	0.112	0.891
Positive	11	1.934	0.949	1.470	1.124	3.816

**Fig. 4:**
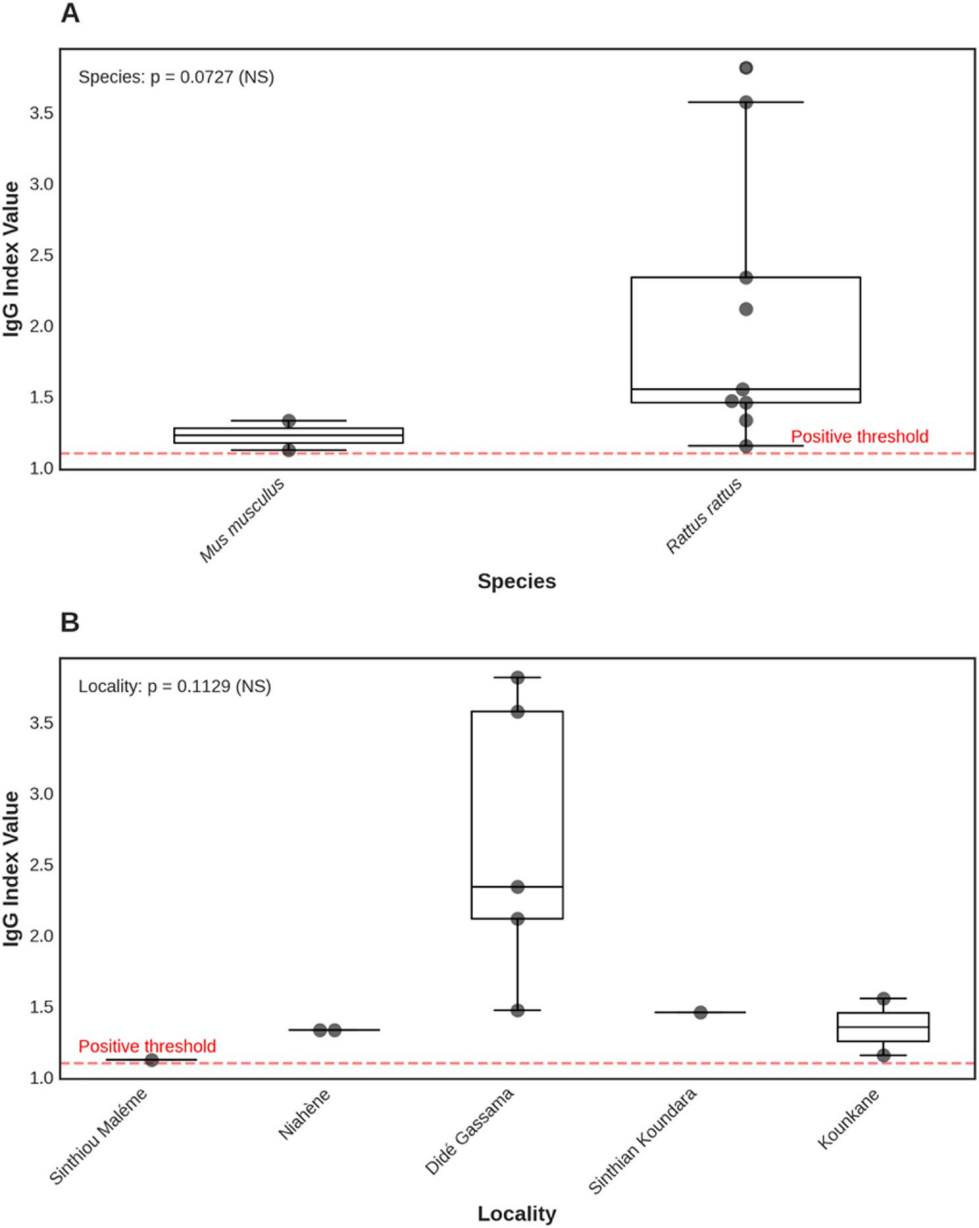
Comparison of LASV-specific IgG responses by species and locality. **(A)** Distribution of LASV IgG index values in *Rattus rattus* and *Mus musculus*. **(B)** Spatial variation in IgG responses across LASV-seropositive localities

Statistical comparisons between exposed species were performed using the Mann–Whitney U test to evaluate differences in LASV-specific IgG responses (Fig. 4A). Although *R. rattus* exhibited a trend toward broader and stronger immune responses compared to *M. musculus* (mean IV: 2.09 ± 0.99 vs. 1.23 ± 0.15), this difference did not reach statistical significance (*p* > 0.05). These findings suggest that the two species may experience comparable levels of exposure or susceptibility, although interpretation is limited by the small sample size, particularly for *M. musculus*. Spatial analysis further indicated that several individuals from Didé Gassama displayed particularly high immune responses (mean IV: 2.66 ± 1) relative to individuals from other localities. However, these geographic differences were also not statistically significant (*p* > 0.05; Fig. 4B).

**Fig. 5:**
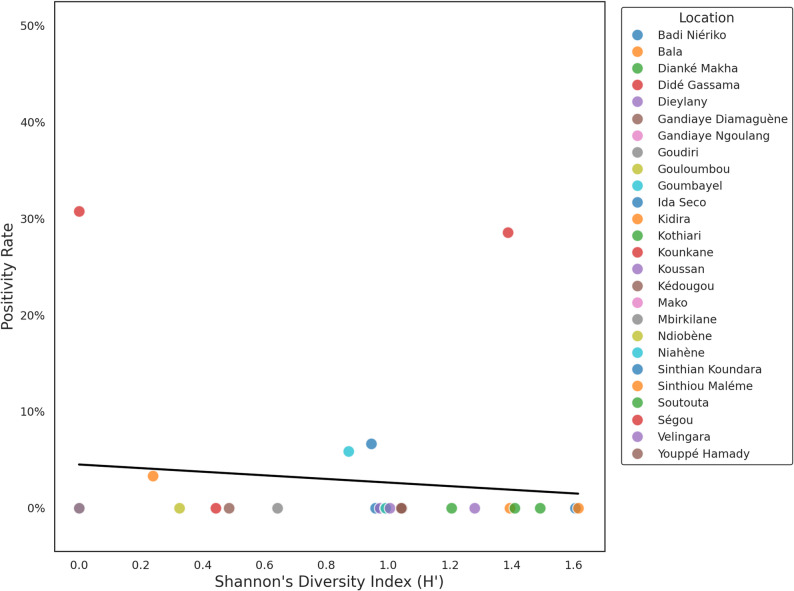
Relationship between rodent community diversity measured by Shannon index and LASV IgG positivity across 26 localities

At the site level, mean Shannon diversity was lower in LASV-positive localities than in LASV-negative localities (mean H’ = 0.69 vs. 0.9), suggesting a possible tendency toward reduced species diversity where LASV exposure was detected (Fig. 5). However, this pattern was weak and not statistically significant. In the diversity–positivity scatterplot, a weakly negative correlation was observed (Pearson r = − 0.12, R² = 0.015, p = 0.556). Likewise, the difference in H’ between LASV-positive and LASV-negative localities was not significant (Mann–Whitney U test, *p* = 0.254). Overall, these results do not provide strong evidence for an association between rodent species diversity and LASV seropositivity at the site level.

## Discussion

Our results show that all IgG-seroreactive animals detected in this study belonged to the commensal species *R. rattus* and *M. musculus*. However, because our evidence is serological and retrospective, these findings should be interpreted cautiously as indicating prior exposure or seroreactivity consistent with LASV exposure, rather than confirmed active infection, reservoir competence, or onward transmission. In this context, the present study does not demonstrate that non-*Mastomys* commensal rodents act as LASV reservoirs in Senegal. Rather, it highlights that LASV-related seroreactivity can be detected in invasive commensal rodent communities and that these exposures occur in focal domestic settings where human–rodent contact is likely to be frequent [11, 16, 17]. We also note that females were more frequently represented among captured *R. rattus* and *M. musculus*, raising the possibility that sex-related differences in exposure or infection dynamics may influence LASV seroreactivity. Vertical transmission or maternal effects could be one explanation, but the present data do not allow this hypothesis to be tested because of the small number of seroreactive animals, the retrospective design, and the absence of age, reproductive, and molecular infection data. This question should therefore be addressed in future prospective studies.

Our data confirm that non-*Mastomys* synanthropes in West Africa can harbor LASV, underscoring their potential as secondary reservoirs or as part of complex transmission webs.

Recent ecological studies in Senegal further confirm that *R. rattus* and *M. musculus* dominate commensal rodent communities in many villages, often displacing native *M. natalensis* populations [18]. Previous work from West Africa has also suggested that such shifts in community composition may modify LASV spillover patterns in some settings [1, 19]. In our dataset, however, the most striking pattern was not a strong association with rodent diversity, but marked heterogeneity in locality-level IgG seropositivity, ranging from 3.6% in Sinthiou Malème to 28.6% in Kounkane, with particularly high values in Didé Gassama (26.3%) and Kounkane (28.6%). This indicates that pooled species-level prevalence masks substantial local variation in LASV seroreactivity. By contrast, the association between rodent community diversity and seropositivity was weak and not statistically supported. Although mean Shannon diversity was lower in LASV-positive than in LASV-negative localities, neither the between-group comparison nor the correlation analysis reached statistical significance. These findings therefore support a focal pattern of LASV exposure and suggest that local ecological or anthropogenic conditions may be more important than diversity alone in shaping exposure risk.

Likewise, experimental and genetic studies suggest that newly invasive *M. musculus* encounters in Senegal may experience altered parasite and immune pressure at recent invasion fronts, which could influence exposure dynamics in newly colonized areas [20].

Environmental and seasonal dynamics further modulate LASV risk across West Africa. Climatic and ecological changes are predicted to alter Mastomys demography and thus human risk. Modeling of Mastomys in West Africa suggests that shifts in the timing or synchrony of the rodent breeding season (driven by climate or land-use change) would change the peak of LASV prevalence in rodents, and hence the timing of human risk [21]. Empirical incidence data in Nigeria support a strong seasonality: spillover to humans surges in the dry season (December–March), coinciding with peak rodent population fluctuations [22]. These ecological insights reinforce that Lassa virus circulation is dynamic, responsive to both natural and anthropogenic pressures.

Our study has limitations that must be acknowledged. Because our rodent data were retrospective and based on serology, we cannot confirm active LASV infection or identify viral lineages. Moreover, the interpretation of the serological findings requires caution. Prior rodent validation is more clearly established for LASV IgG detection in *Mastomys natalensis* and *M. erythroleucus* than in the non-*Mastomys* commensal species represented here, and IgM has not been formally validated in rodents. Accordingly, the absence of IgM reactivity in our study should not be interpreted as excluding recent infection. More broadly, the detection of IgG-seroreactive *R. rattus* and *M. musculus* should be understood as evidence of seroreactivity consistent with prior exposure rather than as proof of active infection, reservoir competence, or onward transmission. Interpretation of the serological signal also requires caution in light of assay performance and host context. Compared with the ELISA validation study of Soubrier et al. [14], the mean IV values observed in our seroreactive commensal rodents were lower than those reported for validated positive *Mastomys* samples, which may reflect weaker or more distant exposure, species-specific assay performance outside the principal validated hosts, or exposure to antigenically related arenaviruses rather than confirmed LASV infection. Accordingly, while locality-level seroreactivity was clearly heterogeneous, the lowest prevalence estimates should be interpreted cautiously, especially when based on only one or two seroreactive animals and in the absence of molecular confirmation.

Future efforts combining molecular detection, viral sequencing, and ecological metadata are needed to clarify reservoir hosts as well as the genetic diversity and temporal dynamics of LASV in Senegal. In addition, although females were more frequently captured in the two seroreactive commensal species, the small number of positive animals and the lack of reproductive or age-stratified data prevented assessment of possible sex-specific transmission mechanisms, including vertical transmission. Additionally, the cross-sectional design cannot capture seasonal fluctuations or direct transmission chains. Nevertheless, strengths include the use of a high-specificity ELISA validated for LASV IgG detection, a relatively extensive geographic coverage, and rigorous species identification. These provide a valuable baseline for integrating rodent surveillance into broader One Health monitoring frameworks. Taken together, these findings provide an updated historical baseline for LASV seroreactivity in Senegalese rodents, while underscoring the need for contemporary surveillance using comparable analytical approaches.

From a public-health and One Health perspective, our findings have several implications. First, the detection of LASV antibodies in Senegalese rodents extends the known ecological range of the virus and underscores the need to maintain vigilance in regions previously considered non-endemic. Building on existing national surveillance systems, closer integration of animal surveillance would support earlier detection of silent circulation. This should include stronger coordination with the environmental sector, notably Senegalese institutions responsible for protected-area management such as the Direction des Parcs Nationaux (DPN), together with national multisectoral coordination mechanisms including Senegal’s national One Health platform under the Haut Conseil national de la sécurité sanitaire mondiale (HCNSSM/One Health), so that human, animal, and environmental health components are more fully represented within a One Health surveillance framework [23]. Second, collaboration with regional and international bodies such as the West African Health Organization (WAHO), the World Health Organization (WHO), Africa CDC, and the World Organization for Animal Health (WOAH) could help harmonize cross-border animal and human surveillance systems. Coordinated serological and genomic monitoring of rodents and other reservoirs would strengthen One Health preparedness for emerging zoonoses in West Africa. Third, linking environmental monitoring, community education, and rodent-control interventions to socioeconomic indicators could mitigate poverty-related exposure risks.

Ultimately, our findings highlight important One Health implications. They suggest that LASV prevention requires integrated surveillance across wildlife, domestic animals and human health sectors. Past reviews emphasize that intensifying interdisciplinary (One Health) efforts is essential to improve LASV surveillance and control in West Africa [24]. In practical terms, this means combining rodent ecology monitoring (including both native and invasive species) with human case surveillance. For public health, understanding where non-*Mastomys* rodents flourish, and how seasonal or environmental changes affect them, can help target interventions (e.g. seasonal sanitation or rodent control) to reduce spillover. Controlling Lassa fever in Senegal and the region will require a coordinated strategy that accounts for shifts in rodent community composition, invasion dynamics, and seasonal ecology. Such a One Health approach, linking animal reservoir studies with clinical surveillance, will be critical to anticipate and mitigate LASV emergence in human populations.

## Electronic Supplementary Material

Below is the link to the electronic supplementary material.

Supplementary Material 1

Supplementary Material 2

Supplementary Material 3

Supplementary Material 4

Supplementary Material 2

Supplementary Material 6

## Data Availability

The datasets generated and analyzed during the current study are available from the corresponding author upon reasonable request.
