# Supplementary figures and images for: Serological evidence of Lassa virus in commensal rodents from Senegal
Source: BMC Infect Dis. 2026 May 2;26:1182. doi: 10.1186/s12879-026-13450-z (PMC13289420; doi:10.1186/s12879-026-13450-z)

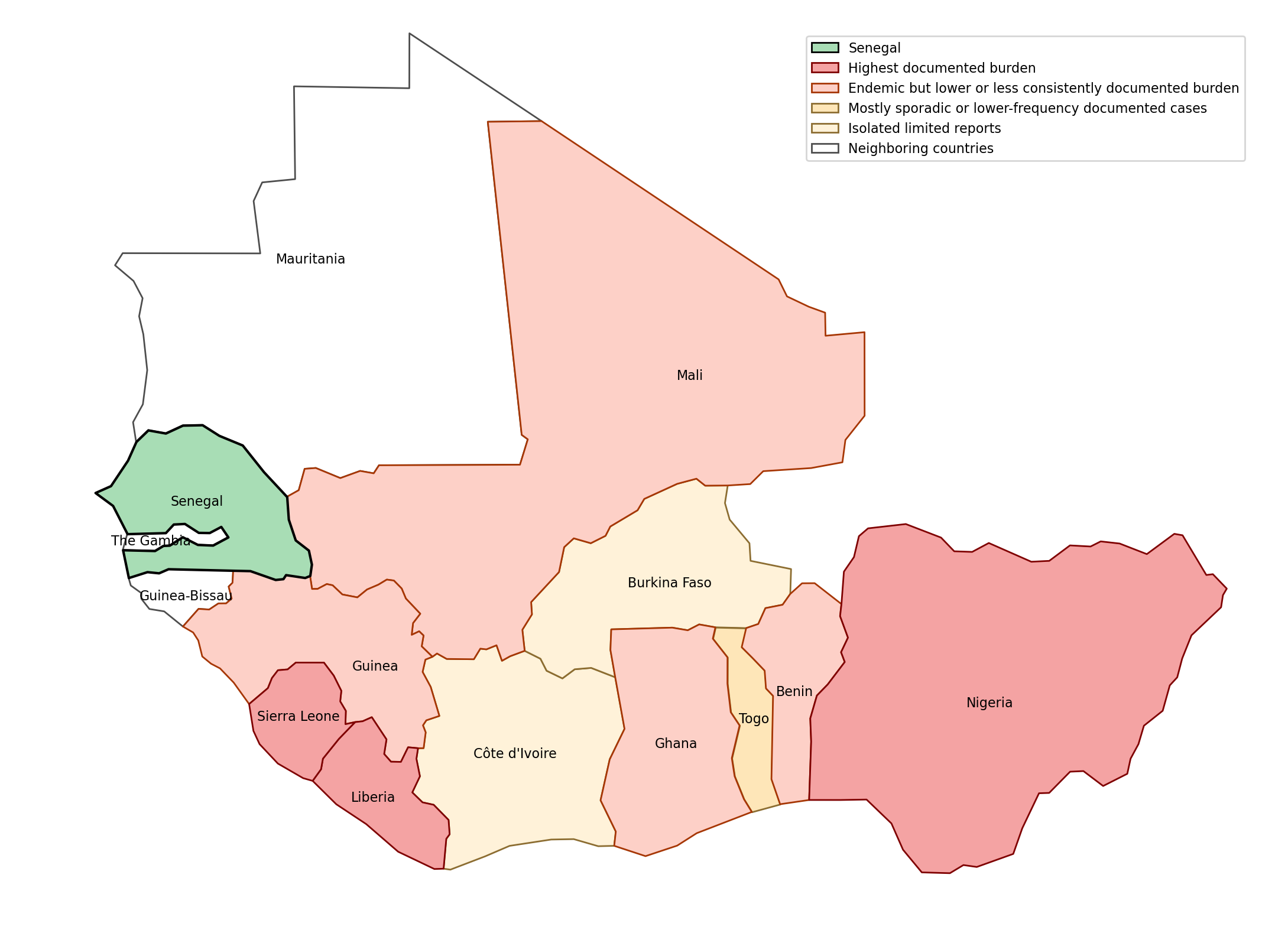

Supplement: Supplementary file 1 — Supplementary Material 1 [file 12879_2026_13450_MOESM1_ESM.png]

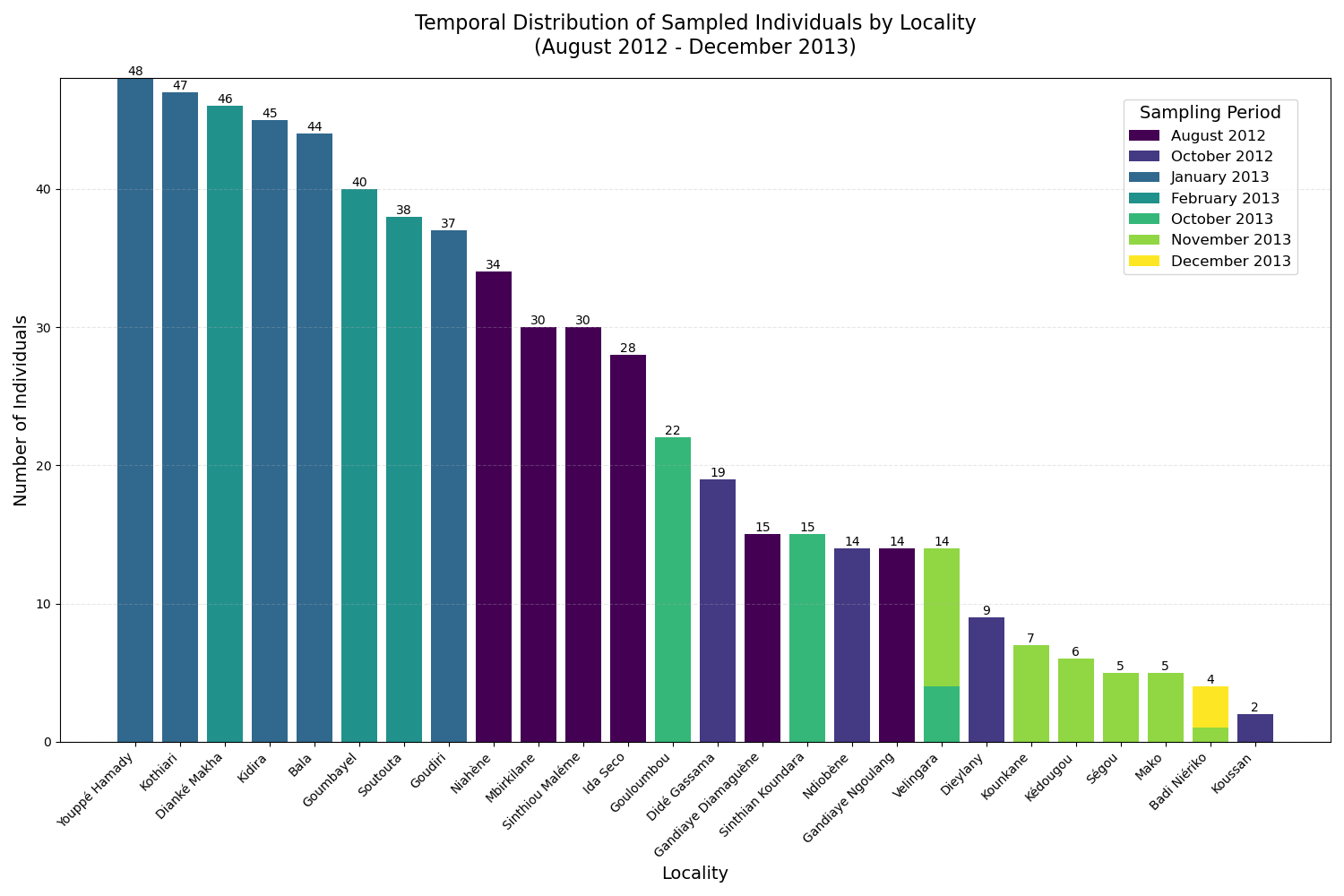

Supplement: Supplementary file 2 — Supplementary Material 2 [file 12879_2026_13450_MOESM2_ESM.png]

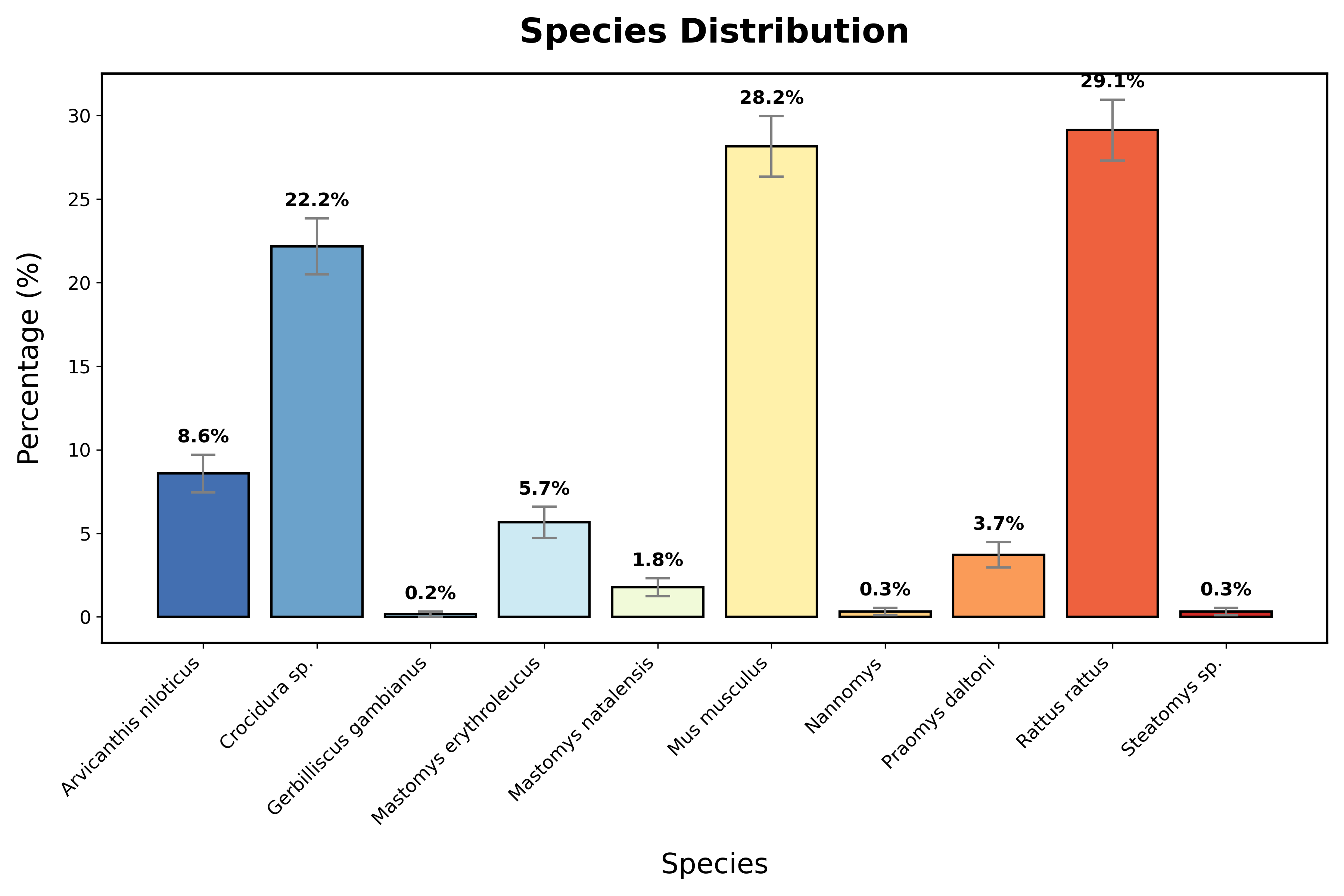

Supplement: Supplementary file 3 — Supplementary Material 3 [file 12879_2026_13450_MOESM3_ESM.png]

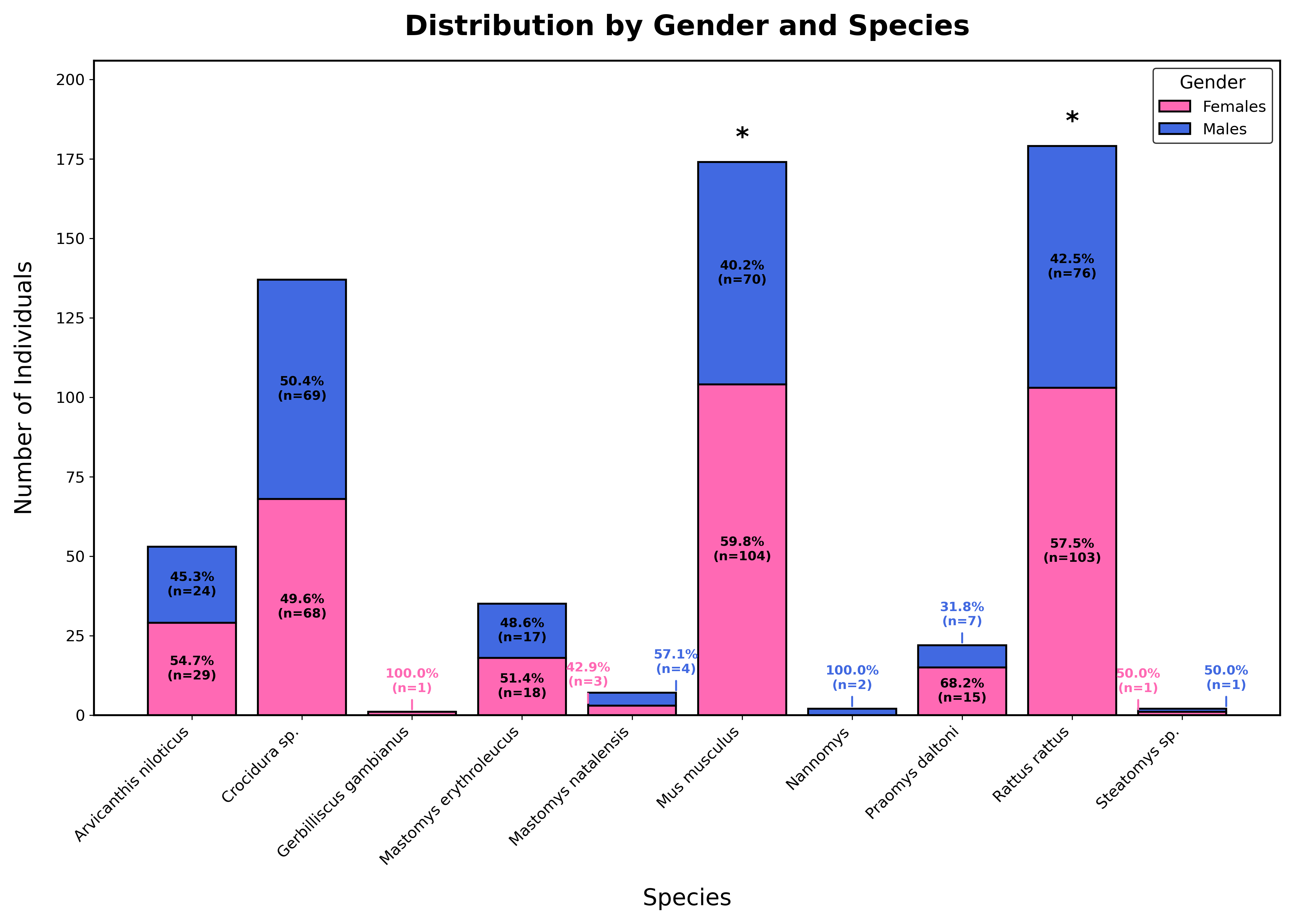

Supplement: Supplementary file 4 — Supplementary Material 4 [file 12879_2026_13450_MOESM4_ESM.png]

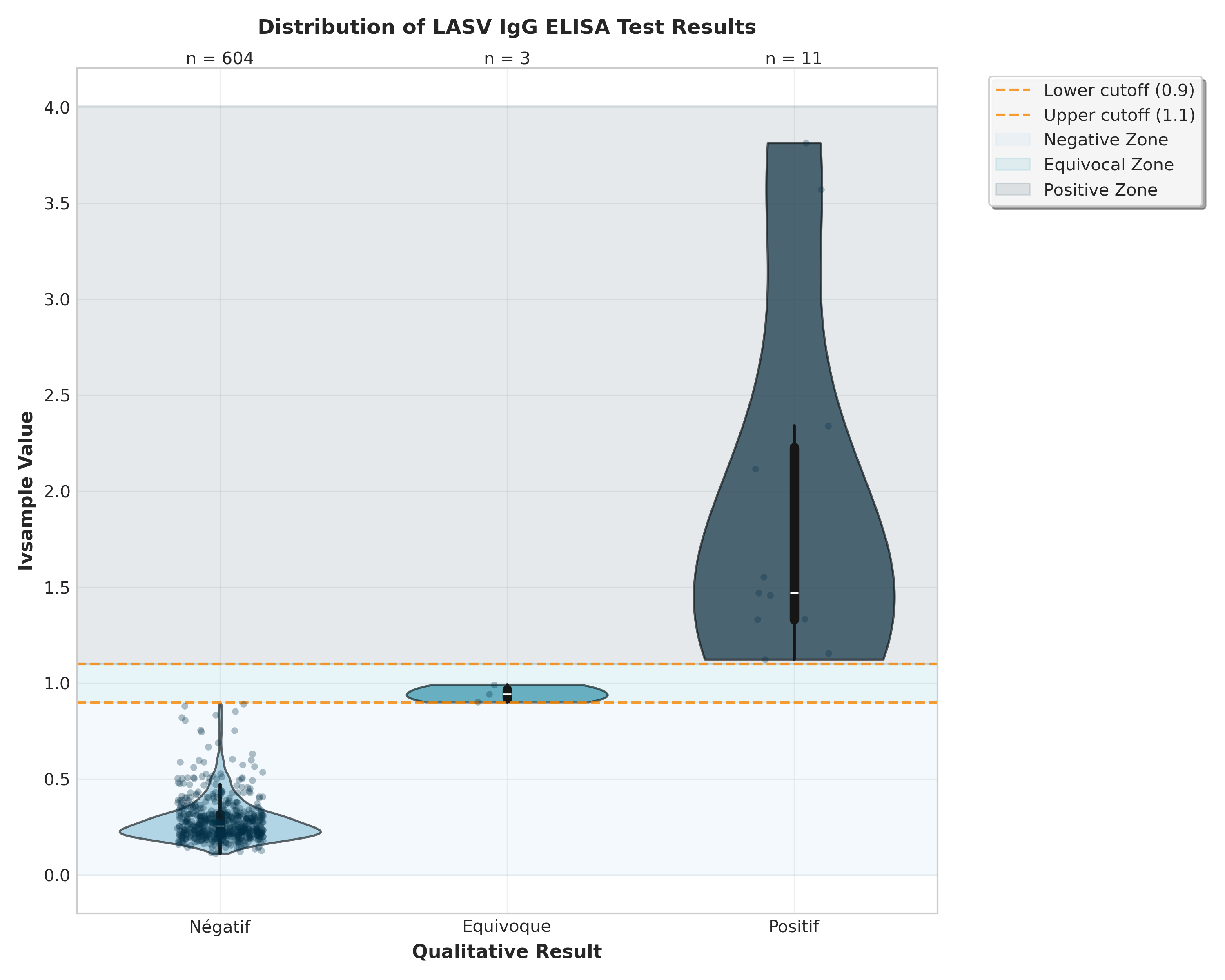

Supplement: Supplementary file 5 — Supplementary Material 2 [file 12879_2026_13450_MOESM5_ESM.png]
