# Supplementary material for: Serological evidence of Lassa virus in commensal rodents from Senegal
Source: BMC Infect Dis. 2026 May 2;26:1182. doi: 10.1186/s12879-026-13450-z (PMC13289420; doi:10.1186/s12879-026-13450-z)
Supplement: Supplementary file 6 — Supplementary Material 6 [file 12879_2026_13450_MOESM6_ESM.docx]

**Supplementary material Legends**

Figure S1. Locator map situating Senegal within West Africa and showing its position relative to neighboring countries where Lassa fever has been documented.

Figure S2. Distribution of rodent captures by locality and sampling period (Senegal, 2012–2013). Bar chart showing the number of individuals captured per site (n = 618), colored by sampling period. The highest sampling intensity occurred in eastern Senegal (Youppe Hamady, Kothiary, Dianké Makha, Kidira, Bala), which together accounted for nearly 37 % of all samples, followed by central sites in Kaffrine (Niahène, Ida Seco) and Kaolack (Gandiaye). Southeastern localities such as Kounkane, Kédougou, and Mako were less represented. Sampling spanned both rainy and dry seasons, capturing ecological contrasts along the trans-Sahelian corridor.

Figure S3. Species composition of rodents captured in Senegal (2012–2013). Bar plot showing the relative percentage of captured individuals by species (n = 618). Commensal species (*Rattus rattus*, 29.1 %; *Mus musculus*, 28.2 %) together accounted for more than half of all captures, followed by *Crocidura* spp. (22.2 %). Other taxa, including *Arvicanthis niloticus*, *Mastomys erythroleucus*, *Praomys daltoni*, and *Mastomys natalensis*—were present at lower frequencies, while *Steatomys* sp., *M*. (*Nannomys) spp*, and *Gerbilliscus gambianus* were rarely detected. Error bars represent standard errors of the mean across sampling localities.

Figure S4. Sex ratio by species among captured rodents (Senegal, 2012–2013).
Stacked bar chart showing the number and proportion of males (blue) and females (pink) within each species. Asterisks denote species with significant female bias (*p* < 0.05). *Rattus rattus* and *Mus musculus* exhibited marked female predominance, whereas *Crocidura sp.*, *Arvicanthis niloticus*, *Mastomys erythroleucus*, and *Mastomys natalensis* showed near-balanced sex ratios. Other species were captured in too few numbers for meaningful statistical comparison.

### **Figure S5. Distribution of LASV IgG ELISA results across qualitative categories.** Violin plot showing the distribution of individual IVsample values according to qualitative ELISA outcomes (negative, equivocal, and positive). Dashed orange lines indicate assay cut-off thresholds (0.9 for negative and 1.1 for positive). Most samples fell within the negative range (n = 604), whereas 11 samples exceeded the positive threshold (IVsample_{sample}sample​ ≈ 1.12–3.82; mean = 1.93 ± 0.95), and three were classified as equivocal (mean = 0.944 ± 0.045). Shaded background zones delineate interpretation ranges defined by the Panadea Diagnostics ELISA protocol.
